# Supplementary material for: Metagenomic binning with assembly graph embeddings
Source: Bioinformatics. 2022 Aug 16;38(19):4481–7. doi: 10.1093/bioinformatics/btac557 (PMC9525014; doi:10.1093/bioinformatics/btac557)
Supplement: btac557_supplementary_data [file btac557_supplementary_data.zip › amber outputs.zip!/amber outputs/simHC/genome/contamination.pdf]

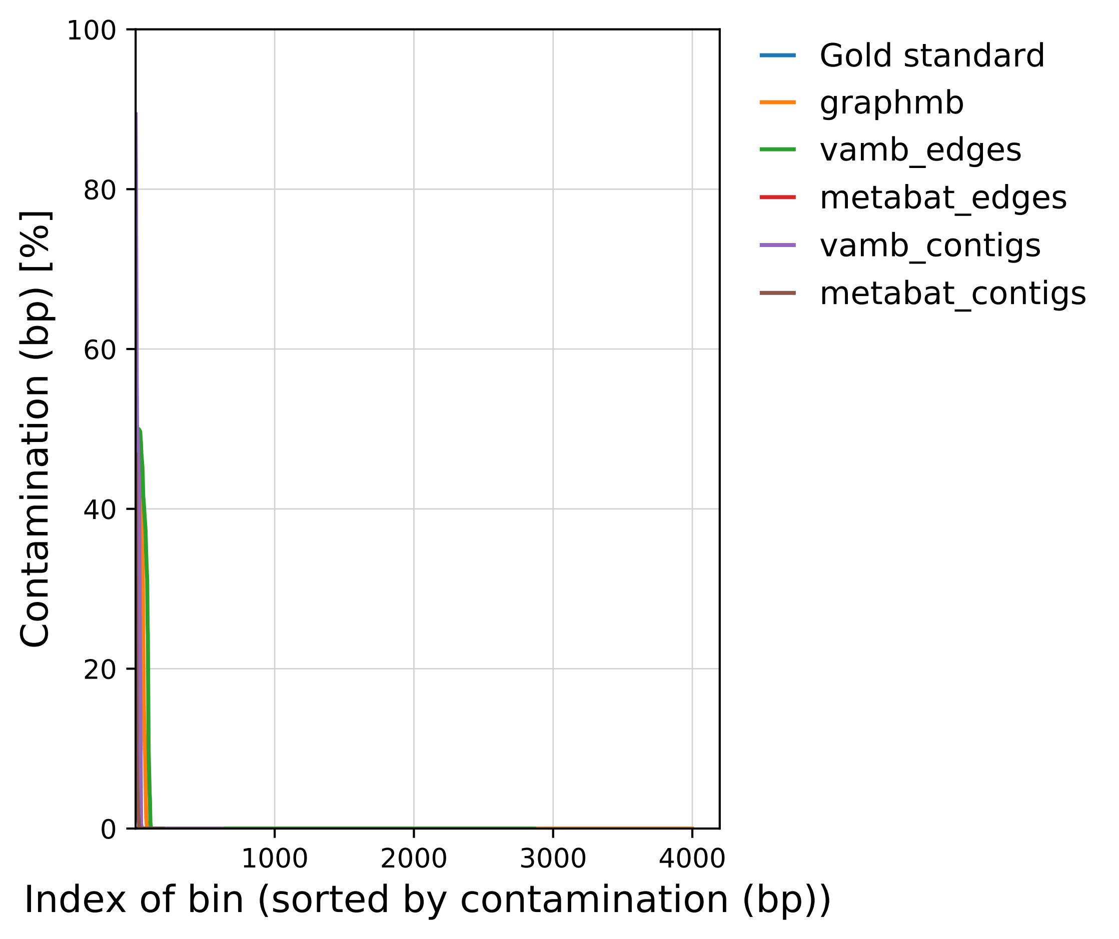

Gold standard
graphmb
vamb_edges
metabat_edges
vamb_contigs
metabat_contigs
Contamination (bp) [%]
100
80
60
40
20
0
1000
2000
3000
4000
Index of bin (sorted by contamination (bp))
